# Unveiling Neuroprotective Potential of Spice Plant-Derived Compounds against Alzheimer's Disease: Insights from Computational Studies

**DOI:** 10.1155/2023/8877757

**Published:** 2023-09-15

**Authors:** Md. Murshid Alom, Rejwana Parvin Bonna, Ariful Islam, Md. Wasim Alom, Md. Ekhtiar Rahman, Md Omar Faruqe, Md. Khalekuzzaman, Rashed Zaman, Md. Asadul Islam

**Affiliations:** ^1^Professor O.I Joarder DNA and Chromosome Research Laboratory, Department of Genetic Engineering and Biotechnology, University of Rajshahi, Rajshahi 6205, Bangladesh; ^2^Department of Genetic Engineering and Biotechnology, University of Rajshahi, Rajshahi 6205, Bangladesh; ^3^Department of Computer Science and Engineering, University of Rajshahi, Rajshahi 6205, Bangladesh

## Abstract

Alzheimer's disease (AD) is a serious threat to the global health care system and is brought on by a series of factors that cause neuronal dysfunction and impairment in memory and cognitive decline. This study investigated the therapeutic potential of phytochemicals that belong to the ten regularly used spice plants, based on their binding affinity with AD-associated proteins. Comprehensive docking studies were performed using AutoDock Vina in PyRx followed by molecular dynamic (MD) simulations using AMBER 14. The docking study of the chosen molecules revealed the binding energies of their interactions with the target proteins, while MD simulations were carried out to verify the steadiness of bound complexes. Through the Lipinski filter and admetSAR analysis, the chosen compounds' pharmacokinetic characteristics and drug likeness were also examined. The pharmacophore mapping study was also done and analyzed for best selected molecules. Additionally, principal component analysis (PCA) was used to examine how the general motion of the protein changed. The results showed quercetin and myricetin to be potential inhibitors of AChE and alpha-amyrin and beta-chlorogenin to be potential inhibitors of BuChE, exhibiting best binding energies comparable to those of donepezil, used as a positive control. The multiple descriptors from the simulation study, root mean square deviation (RMSD), root mean square fluctuation (RMSF), hydrogen bond, radius of gyration (Rg), and solvent-accessible surface areas (SASA), confirm the stable nature of the protein-ligand complexes. Molecular mechanic Poisson-Boltzmann surface area (MM-PBSA) binding free energy calculations indicated the energetically favorable binding of the ligands to the protein. Finally, according to pharmacokinetic properties and drug likeness, characteristics showed that quercetin and myricetin for AChE and alpha-amyrin and beta-chlorogenin for BuChE were found to be the most effective agents for treating the AD.

## 1. Introduction

Alzheimer's disease (AD) is a progressive neurological disorder that is the most common cause of dementia [1], affecting at least 27 million individuals and accounting for 60 to 70% of all dementia cases [2]. It is considered progressive because the symptoms worsen over time [3]. In its most advanced phases, this disease leads patients to lose cognitive function, brain cells, and memory, leaving them completely dependent on others for survival [4]. A comprehensive treatment for this condition has not been found after more than a century of research and disease identification [5].

Cholinergic neurotransmission plays a crucial role in impaired cognitive function, which is a hallmark of AD as well as other forms of dementia [6]. There were treatments proposed to inhibit accumulation of amyloid-*β*, tau hyperphosphorylation, and immunotherapy; however, these treatments failed to provide effects and were consequently abstained in phase II or III clinical trials [7]. Currently, the primary method for treating the cognitive and behavioral symptoms of mild-to-moderate stages of AD involves enhancing cholinergic neurotransmission [8]. Therefore, acetylcholinesterase (AChE) and butyrylcholinesterase (BuChE) have become a major focus of AD research in recent decades [9]. Two cholinesterases, AChE and BuChE, hydrolyze acetylcholine (ACh) in the brain; AChE is more prevalent than BuChE in the brain tissue of AD patients, which leads to the breakdown of ACh in the hippocampus and cerebral cortex [10]. During AD progression, AChE activity in the temporal lobe and hippocampus is lowered by 67% relative to normal levels, while BuChE activity increases to 165% of normal levels [11]. AChE and BuChE (cholinesterase) inhibitors (ChE-Is) prevent the breakdown of the neurotransmitter by boosting the amounts of ACh in the brain, hence strengthening the inadequate cholinergic neurotransmission in the brain. Both AChE and BuChE have been implicated in the etiology of AD, and studies have demonstrated the therapeutic value of inhibiting both enzymes [10, 12].

Tacrine is a reversible AChE and BuChE inhibitor which was the first compound to enter clinical trials as a potential AD treatment showing significant benefits on memory performance in young and elderly normal subjects [13]. Donepezil, a novel AChE inhibitor produced from indanone, was discovered in 1983 by Sugimoto et al. at Japan's Eisai Research Laboratory [14]. The FDA first approved donepezil in 1996 for mild-to-moderate AD, and in 2010, they expanded its use to moderate-to-severe AD at the level of 23 mg/day [15]. Rivastigmine, another FDA-approved molecule, is an irreversible AChE and BuChE inhibitor for the treatment of mild-to-moderate AD [16]. In 2000, a reversible AChE inhibitor, galantamine, was approved as a symptomatic medication for AD which modifies the allosteric site of the nicotinic cholinergic receptors [17]. These inhibitors disrupt the effects of AChE at the synapse, resulting in anorexia, diarrhea, nausea, vomiting, and bradycardia [18]. These molecules do not cure AD, but they may improve memory, awareness, and functional capacity. Without a cure for the condition, researchers continue to look for a treatment.

With this motive to forage for potential therapeutics, the computer-aided *in silico* technique has been broadly used for the early phases of drug development. Computer-aided rational drug design is entrenched by the inspection of the structures and functions of biological targets to find new medications [19]. It is a useful technique of predicting the potential drug candidates for diverse diseases in a cost-effective and time-efficient manner that minimizes mistakes in the final phases [20, 21]. A rapid *in silico* approach has been exercised to find and identify new natural product that leads against AChE and BuChE as therapeutic targets based on the three-dimensional structure of receptor [22]. Concerning this, the information of protein-ligand interactions was harnessed by utilizing the X-ray crystal structures of AChE and BuChE to decipher valuable insights into structural features that are critical for inhibitor binding. Here, we amassed a total of 1701 bioactive compounds from ten daily used spice plants as ligands. Research on medicinal plants will persist as long as humanity endures, given their significant contributions to disease treatment over numerous years [23, 24]. Molecular docking was performed to predict receptor-ligand interaction at the molecular mechanism of action, allowing prospective therapeutic candidates to be identified in a relatively short amount of time [25]. To validate the predictions from docking investigations, MD simulation has been intensively used, which is a computer simulation tool for studying the physical motions of atoms and molecules [26]. Molecular mechanic Poisson-Boltzmann surface area (MM-PBSA) is adopted to calculate ligand-macromolecule interaction free energies which are extensively used in the study of biomolecular interactions [27]. This study also employed ADME/TOX to evaluate the drug likeness of screened results from docking studies. Aforementioned tools might enhance the prediction of binding affinity and stability of the receptor ligand complexes, which aids in the identification of lead compounds.

**Figure 1:**
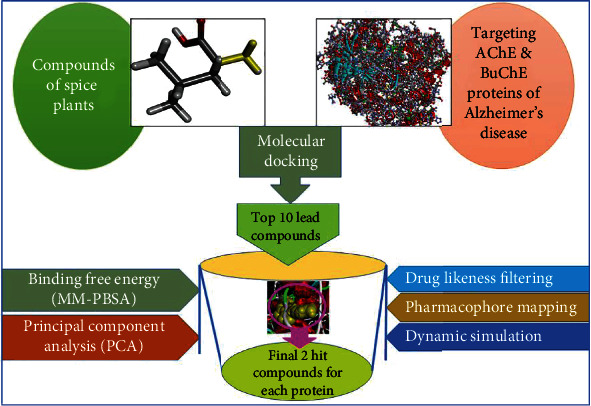
Graphical representation of this study.

Natural sources of phytochemicals such as plants are being studied for drug development, as they are less poisonous and have fewer side effects than synthetically produced molecules [28]. Hence, in this study, we aimed to design likely inhibitors from the phytochemicals of medicinal spice plants against the potential drug targets AChE and BuChE of AD. The workflow of this study is presented in Figure 1.

## 2. Materials and Methods

### 2.1. Protein Retrieval and Preparation

The target proteins, i.e., AChE (PDB ID: 4EY6) [29] and BuChE (PDB ID: 1P0I) [30], were retrieved from the Protein Data Bank (PDB) (https://www.rcsb.org/) database maintained by Research Collaboratory for Structural Bioinformatics (RCSB). The two PDB IDs were considered due to their X-ray crystallographic structure, lower resolution (<2.5 Å), and percentile scores in global validation metrics which indicates the better structure quality. The protein structures were preprocessed through PyMOL software (version 2.5) [31]. This operation cleaned and optimized the protein models by removing the native inhibitors as well as other heteroatoms including water molecules. To make proteins protonated for enhanced docking performance, hydrogen atoms have been added to them. The preprocessed macromolecules were then optimized, verified, and energy-minimized using the Swiss-PDB Viewer [32].

### 2.2. Ligand Retrieval and Preparation

A total of 1701 bioactive compounds from the ten daily used spice plants including *Allium cepa* (onion), *Allium sativum* (garlic), *Zingiber officinale* (ginger), *Capsicum annuum* (chili), *Cinnamomum verum* (cinnamon), *Laurus nobilis* (bay leaf), *Curcuma longa* (turmeric), *Cuminum cyminum* (cumin), *Syzygium aromaticum* (clove), and *Coriandrum sativum* (coriander) were selected as ligands. Bioactive compound's library was prepared from Dr. Duke's Phytochemical and Ethnobotanical Databases (https://phytochem.nal.usda.gov/phytochem/search). 3D structures of these compounds were obtained from PubChem database (https://pubchem.ncbi.nlm.nih.gov/) in .sdf format. The structures of ligands were prepared using the Open Babel [33] software that is included as a default option in the PyRx.

### 2.3. Molecular Docking

Molecular docking was carried out by PyRx software (version 0.8) [34] to explore all possible orientations, conformations, and binding affinities for the ligands with AChE and BuChE active site. All ligands were converted to PDBQT format using Open Babel software [33] to prepare them in an admissible format for docking in AutoDock Vina. The grid box was generated by entrapping the whole protein into the box to implement blind docking. Molecular visualization of the docking results was performed, and nonbonding interactions between the docked protein-ligand complexes and the docking pose were analyzed by using BIOVIA Discovery Studio Client 2021 [35]. Conformation indicating the lowest docking score (in kcal/mol) was selected as lead compound.

### 2.4. ADME and Toxicity Analysis

SwissADME [36] and admetSAR [37] servers were used with the intention to estimate the pharmacological and pharmacokinetic characteristics of selected lead compounds applying Lipinski's rule of five (RO5). Canonical simplified molecular-input line-entry system (SMILES) structures were retrieved for each lead molecule from PubChem database. These SMILES are required as an entry system for these servers in order to predict the drug likeness of lead compounds.

### 2.5. Pharmacophore Mapping

Pharmacophore modeling helps to validate structure-activity relationship (SAR) findings and guide the design of new compounds with optimized activity and improved target selectivity. The pharmacophore mapping study of the two best ligands was carried out by online server PharmMapper (http://www.lilab-ecust.cn/pharmmapper/). The ligands were downloaded in .sdf format from PubChem server and afterwards uploaded, and the “maximum number of conformation” parameter was set at 1000. All possible targets were kept at the “select target set” parameter, and the “number of reserved matched target” parameter was kept 1000. The cut-off value for the fit score in the advanced settings was set at 0. The default settings were used for all other parameters. The pharmacophore mapping experiment was done by utilizing two best ligand molecules for each protein among the 10 selected lead compounds.

### 2.6. Molecular Dynamic Simulation

The molecular dynamic simulation was done in YASARA dynamics [38] using the AMBER 14 force field [39]. Complexes were adjusted, hydrogen bond networks were oriented, and the cubic simulation cell was constructed. In order to minimize the protein complexes using a TIP3P water solvation model, the steepest gradient techniques were used with a simulated annealing strategy (0.997 g/L1, 25 c, and 1 atm) [40]. At 310 K, 0.9% NaCl, and pH 7.4, the simulated system was neutralized [41]. The particle mesh Ewald method was used to determine the electrostatic interaction, with a cut-off radius of 8 Å. In order to allow the protein to travel freely, the simulation cell was stretched to 20 Å on both sides of the system. The simulation temperature was kept consistent using a Berendsen thermostat [42]. The simulation was run at 1.25 frames per second with the trajectories saved every 100 ps and carried out for over 100 ns. Lastly, simulation snapshots were used to determine the root mean square deviation (RMSD), the radius of gyration (Rg), solvent-accessible surface area (SASA), hydrogen bonds, and root mean square fluctuation (RMSF). Subsequent trajectory analyses were implemented by SciDAVis software available at http://scidavis.sourceforge.net.

### 2.7. Binding Free Energy Calculation Using MM/PBSA

Calculations of the binding free energy are an important method to assess the degree of drug binding to a protein in order to analyze the energetics and stability of the protein-drug complex. All snapshots were then subjected to YASARA software's MM-Poisson-Boltzmann surface area (MM-PBSA) binding free energy (BFE) using the following formula: BFE = EpotReceptor + EsolvReceptor + EpotLigand + EsolvLigand − EpotComplex − EsolvComplex. In this case, built-in YASARA macros were used with AMBER 14 as the force field to determine the MM-PBSA binding energy.

### 2.8. Principal Component Analysis (PCA)

Principal component analysis (PCA) was carried out to highlight the variance and uniformity in the profile of molecular dynamic simulation profile data. It is possible to categorize structural alterations in protein-ligand complexes that occur throughout the course of a molecular simulation by contrasting different variables. By diagonalizing the matrices and solving the eigenvalue and eigenvector problems for the covariance matrices, PCA of the complexes was obtained. Eigenvalues reflected structural fluctuational amplitude and direction, whereas eigenvectors represented structural fluctuational direction [43, 44]. Preprocesses from MD trajectories of 100 ns were used; then, the feature was standardized by scaling to unit variance and removing the mean. scikit-learn v1.2.2 package was used to calculate the variance ratio in Python program.

## 3. Results

### 3.1. Molecular Docking

The AChE and BuChE targets were successfully docked with each of the selected ligand molecules from ten daily used spice plants. Because lower binding energies refer to higher binding affinities, the ligand molecules with the lowest binding energies or docking scores were regarded as the most effective ligand molecules for blocking the target receptor [45, 46].

By analyzing the docking interactions for AChE protein, we found that Fmoc-His(Trt)-Wang resin, cucurbitaxanthin A, (3R,3′R,15-cis)-b,b-carotene-3,3′-diol, epsilon-carotene, quercetin, quercetin-3′-glucuronide, myricetin, folic acid, quercetin 3-O-glucuronide, and luteolin exhibited the lowest binding affinity (best binding score) of -12.2, -12, -11.4, -10.8, -10.6, -10.6, -10.6, -10.5, -10.5, -10.5, and -10.4 kcal/mol, respectively.

In the case of BuChE protein, proanthocyanidin A2, procyanidin B1, alpha-amyrin, strictinin, proanthocyanidin B2, Fmoc-His(Trt)-Wang resin, beta-chlorogenin, beta-amyrin, oleanolic acid, and coriandrinonediol exhibited the lowest binding affinity (best binding score) of -12.2, -12.2, -12.1, -12.1, -12, -11.8, -11.2, -11.2, -11.1, and -11.1 kcal/mol, respectively. Donepezil as a control drug showed -7.6 kcal/mol binding affinity with AChE protein and -9.8 kcal/mol with BuChE protein (Table 1).

After performing the molecular docking, this study analyzes the drug likeness, ADME, and pharmacophore properties, and the two phytochemicals, namely, quercetin and myricetin, were selected as the probable inhibitor of AChE protein. In another case, the two phytochemicals, namely, alpha-amyrin and beta-chlorogenin, were selected as the probable inhibitor of the BuChE protein.

**Figure 2:**
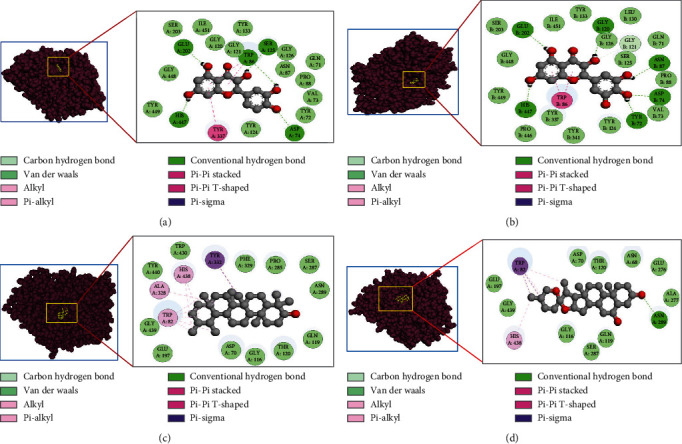
Predicted pose from the docking analysis showed binding mode of compounds within the active site of targets. The 3D positioning of ligand within binding sites of the protein was shown on the surface view, and the electrostatic interactions of corresponding protein-ligand complexes were shown on the 2D view. Here, a and b indicate the interactions of acetylcholinesterase_quercetin and acetylcholinesterase_myricetin complexes. On the other hand, c and d indicate the interactions of butyrylcholinesterase_*α*-amyrin and butyrylcholinesterase_*β*-chlorogenin complexes.

In the interaction of AChE protein, the quercetin formed van der Waals bond with GLN71, TYR72, VAL73, ASN87, PRO88, GLY120, GLY121, TYR124, GLY126, TYR133, SER203, GLY448, TYR449, and ILE451; conventional hydrogen bond with ASP74, TRP86, SER125, GLU202, and HIS447; and pi-pi stacked and pi-pi T-shaped with TYR337 amino acid residues (Figure 2(a)). Followed by the top interaction of AChE protein, the myricetin formed van der Waals bond with GLN71, VAL73, PRO88, TYR124, SER125, GLY126, LEU130, TYR133, SER203, TYR337, TYR341, PRO446, ILE451, GLY448, and TYR449; conventional hydrogen bond with TYR72, ASP74, ASN87, GLY120, GLU202, and HIS447; a carbon-hydrogen bond with GLY121; and two pi-pi stacked bond with TRP86 amino acid residues (Figure 2(b)).

In the case of BuChE protein, the alpha-amyrin formed van der Waals bond with ASP70, GLY116, GLN119, THR120, GLU197, PRO285, SER287, ASN289, PHE329, TRP430, TYR440, and GLY439, a pi-sigma bond with TYR332, and alkyl and pi-alkyl bond with TRP82, ALA328, and HIS438 amino acid residues (Figure 2(c)). Further, followed by the top interaction of BuChE protein, the beta-chlorogenin formed van der Waals bond with ASN68, ASP70, GLY116, GLN119, THR120, GLU197, GLU276, ALA277, SER287, and GLY439, a conventional hydrogen bond with ASN289, two pi-sigma bonds with TRP82, and pi-alkyl bond with TRP82 and HIS438 amino acid residues (Figure 2(d)).

**Figure 3:**
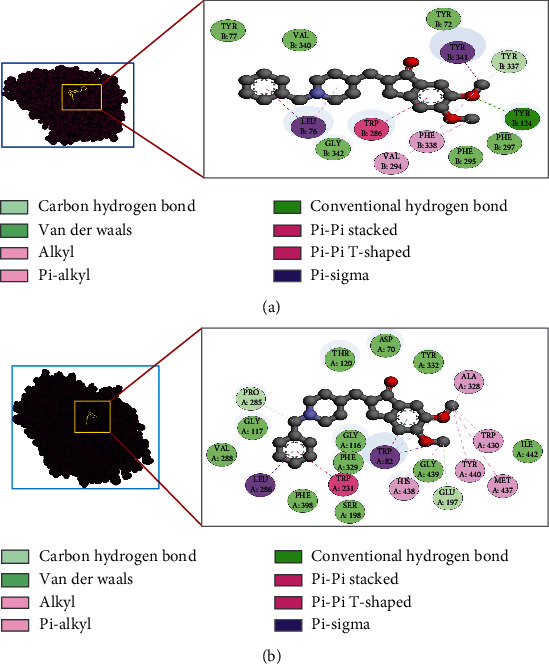
Predicted pose from the docking analysis showed binding mode of compounds within the active site of targets. The 3D positioning of ligand within binding sites of the protein was shown on surface view, and the electrostatic interactions of corresponding protein-ligand complexes were shown on 2D view panel. Here, a and b indicate the interactions of acetylcholinesterase_donepezil and butyrylcholinesterase_donepezil complexes, respectively.

Furthermore, this study also considers the donepezil as a positive control for AChE protein where donepezil formed van der Waals bond with TYR72, TYR77, PHE295, PHE297, VAL340, and GLY342, a conventional hydrogen bond with TYR124, carbon hydrogen bond with TYR337, pi-sigma bond with LEU76 and TYR341, pi-pi stacked bond with TRP286, and alkyl and pi-alkyl bond with VAL294 and PHE338 amino acid residues (Figure 3(a)).

Followed by the interaction for BuChE protein, donepezil formed van der Waals bond with ASP70, GLY116, GLY117, THR120, SER198, VAL288, PHE329, TYR332, PHE398 GLY439, and ILE442, carbon hydrogen bond with GLU197 and PRO285, pi-sigma with TRP82 and LEU286, pi-pi stacked and pi-pi T-shaped bond with TRP231, and alkyl and pi-alkyl bond with ALA328, TRP430, MET437, HIS438, and TYR440 amino acid residues (Figure 3(b)).

### 3.2. ADME/TOX Analysis

The different pharmacokinetic parameters of the discovered phytochemicals were assessed using Lipinski's rule of five. After molecular docking, this study selected the top 10 compounds for ADME analysis in the case of both targeted proteins, AChE and BuChE. Quercetin and myricetin for AChE protein, as well as alpha-amyrin and beta-chlorogenin compounds for BuChE protein, confirmed with Lipinski's rule of five, and ADME/TOX may demonstrate optimal drug-like behavior. Tables 2 and 3 display the findings of the ADME/TOX analysis, which looks at the compound's absorption, distribution, metabolism, and excretion/toxicity. Based on the lowest binding affinity, electrostatic bond formation, and pharmacokinetic properties, the best two ligands were considered for conducting the further study.

### 3.3. Pharmacophore Mapping

In the case of AChE, quercetin and myricetin gave almost similar fit scores of 2.97 and 2.986, respectively, in the experiment of pharmacophore mapping. Quercetin showed a normalized fit score of 0.7425 and myricetin generated a normalized fit score of 0.7464. Furthermore, both quercetin and myricetin generated hydrophobic centers of 2, and also, each showed 2 hydrogen bond donors. Moreover, none of the molecules showed a positively charged center, negatively charged center, aromatic ring, and hydrogen bond acceptor.

**Figure 4:**
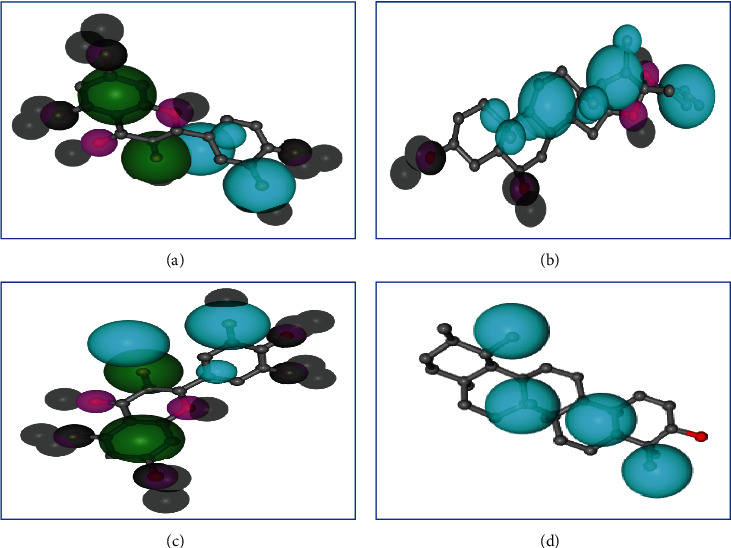
Pharmacophore mapping of (a) quercetin, (b) myricetin, (c) *α*-amyrin, and (d) *β*-chlorogenin. Here, light blue color, hydrophobic center; green color, hydrogen bond donor; pink color, hydrogen bond acceptor; and gray, excluded volumes of the binding pocket.

In another case of BuChE, alpha-amyrin and beta-chlorogenin showed a fit score of 4 and 3, respectively, and showed a normalized fit score of 1 for both alpha-amyrin and beta-chlorogenin. Alpha-amyrin showed hydrophobic centers of 4, and beta-chlorogenin generated hydrophobic centers of 3. Moreover, none of the molecules showed a positively charged center, negatively charged center, aromatic ring, hydrogen bond donor, and hydrogen bond acceptor (Table 4 and Figure 4).

### 3.4. Molecular Dynamic Simulation

After a virtual screening, quercetin and myricetin for AChE as well as alpha-amyrin and beta-chlorogenin for BuChE were picked out of the drug candidates for the dynamic simulation analysis. MD simulations were run on the YASARA structural tool version 20.12.24.W.64 (using the AMBER 14 force field) with a 100 ns time step to investigate the dynamic activity of the protein-ligand complex in a solvent environment over time.

**Figure 5:**
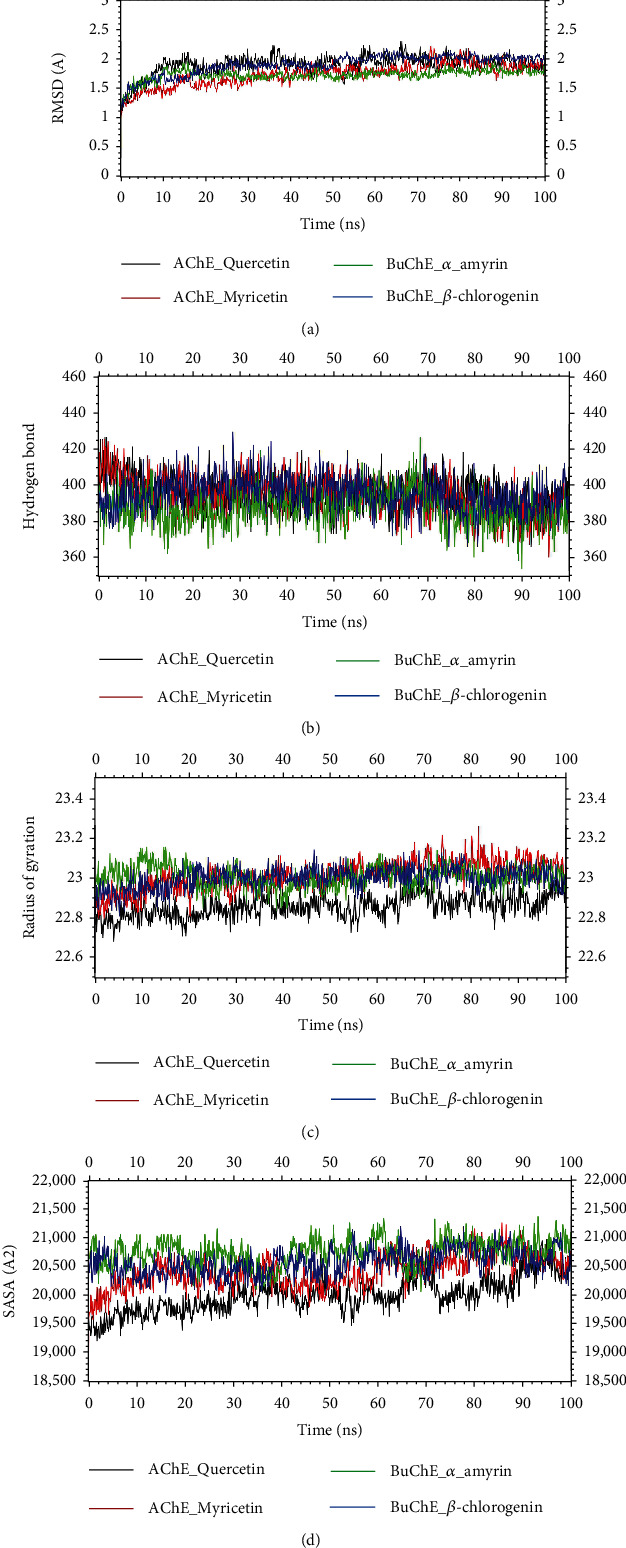
Analysis of the structural changes of protein-ligand complexes during 100 ns MD simulation runtime. Here, (a) root mean square deviations (RMSD), (b) hydrogen bond, (c) radius of gyration, and (d) solvent accessible surface area (SASA). Here, black, red, green, and blue lines denote acetylcholinesterase_quercetin, acetylcholinesterase_myricetin, butyrylcholinesterase_*α*-amyrin, and butyrylcholinesterase_*β*-chlorogenin complexes, respectively. Each analysis technique characterized the structural changes and differences among the various protein complexes studied.

In order to assess the stability and rigidity of the complex over the course of the simulation, the RMSD of the docked complex was evaluated in relation to the beginning structure, as shown in Figure 5(a).

Figure 5(a) represents the RMSD of the AChE_quercetin, AChE_myricetin, BuChE_alpha-amyrin, and BuChE_beta-chlorogenin complexes. AChE_quercetin and AChE_myricetin complexes showed the RMSD around 1.5 Å to 2.2 Å and 1.3 Å to 2.2 Å, respectively. The four docked complexes had initial upper trend of the RMSD profile, but they did not deviate that too much. Most importantly, during the early simulation phase, all four complexes reached steady state. BuChE_alpha-amyrin and BuChE_beta-chlorogenin complexes showed the RMSD around 1.2 Å to 2.0 Å and 1.2 Å to 2.1 Å, respectively. The average RMSD for the AChE_quercetin, AChE_myricetin, BuChE_alpha-amyrin, and BuChE_beta-chlorogenin complexes are 0.6 Å, 0.9 Å, 0.8 Å, and 0.9 Å, respectively.

The number of hydrogen bonds was also used to assess the protein stability and folding success. For AChE_quercetin complex, the hydrogen bond analysis revealed an increasing number of hydrogen bonds with respect to time over the course of the simulations (Figure 5(b)). Only a little bit fluctuation occurred at 91 ns. Although the number of hydrogen bonds fluctuated during simulation, the protein's stability remained constant. In the case of AChE_myricetin complex, an increasing number of hydrogen bonds were visible, but at 95 ns, a considerable fluctuation occurred, and subsequently, it indicates the steady state of protein. BuChE_alpha-amyrin complex showed the increased number of hydrogen bonds and at 89 ns displayed a lowered degree of fluctuation. But overall, the protein's steadiness stayed constant. Finally, BuChE_beta-chlorogenin complex revealed a cumulative number of hydrogen bonds during the simulations. After 74, 78, and 80 ns, it exhibited a substantial fluctuation and remains constant stability of the complex.

Figure 5(c) depicts the radius of gyration for four simulated protein complexes. The radius of gyration is calculated using the protein's center of mass, which indicates how compact the protein structure is. It will stay constant if the protein is stable; however, it will change over time due to instability. Importantly, AChE_quercetin, AChE_myricetin, BuChE_alpha-amyrin, and BuChE_beta-chlorogenin complexes showed little fluctuations in our study, subsequently indicating their stability over time. The average fluctuations in AChE_quercetin, AChE_myricetin, BuChE_alpha-amyrin, and BuChE_beta-chlorogenin complexes were 0.3 Å, 0.5 Å, 0.4 Å, and 0.3 Å, respectively.

Additionally, the SASA or solvent-accessible surface areas of the four complexes were evaluated to see if the protein surface or volume had changed. In this study, the total SASA was calculated which indicates the biomolecular surface area is accessible to solvent molecules. This surface is made up of all the spots that the water probe's center can touch as it rolls over the solute. The longer protein volume is correlated with a greater SASA profile, whereas the shorter protein volume is correlated with a lower SASA profile. The SASA profile for all complexes initially had a higher profile, but AChE_quercetin complex after 50 ns showed lower profile, AChE_myricetin complex exhibited stable profile during simulation, BuChE_alpha-amyrin complex showed lower profile after 35 ns and 65 ns, and BuChE_beta-chlorogenin complex displayed a little bit lowered SASA profile (Figure 5(d)).

**Figure 6:**
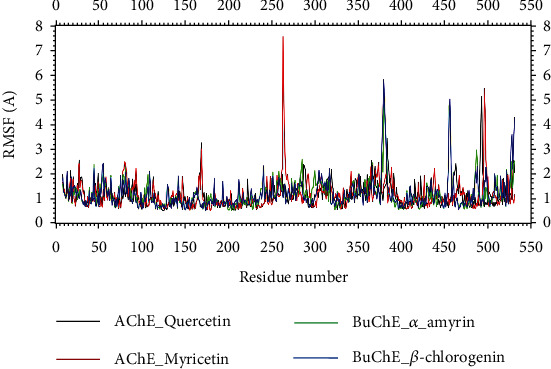
Root mean square fluctuation (RMSF) of the amino acid residues from the complexes. Here, black, red, green, and blue lines denote quercetin_acetylcholinesterase, myricetin_acetylcholinesterase, *α*-amyrin_butyrylcholinesterase, and *β*-chlorogenin_butyrylcholinesterase complexes, respectively.

Through RMSF descriptors, the flexibility of the protein-compound system across amino acid residues can be assessed. Lower RMSF values were found for all of the complexes and the corresponding amino acid residues, suggesting decreased flexibility. The maximum amino acid residues had lower RMSF profile from below 2.5 Å except ASP494, ARG493, ALA542, TRP385, ARG165, SER541, LYS538 (for AChE_quercetin complex), THR262, PRO495, GLY263, ALA542, LYS496, SER541, ARG165, LYS538, ARG534, GLY264 (for AChE_myricetin complex), ASP454, VAL377, ARG453, GLN380, ASN485, ASP375 (for BuChE_alpha-amyrin complex), VAL377, ASP454, TRP376, VAL529, ARG453, PHE526, LYS528, PHE525, ASP375, and GLN380 (for BuChE_beta-chlorogenin complex) amino acid residues Figure 6.

### 3.5. Binding Free Energies of Interactions

**Figure 7:**
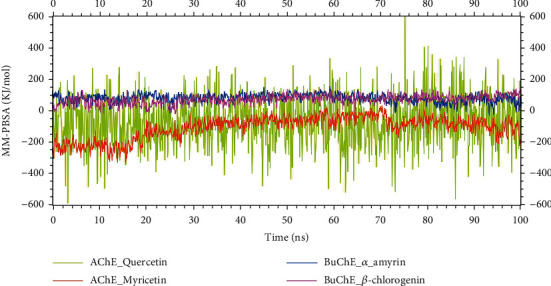
The binding free energy (kJ/mol) of each complex was determined through a 100 ns molecular dynamic simulation based on MM-PBSA, showing the change in binding stability of each complex throughout the simulation. Here, light green, orange, blue, and pink lines denote quercetin_acetylcholinesterase, myricetin_acetylcholinesterase, *α*-amyrin_butyrylcholinesterase, and *β*-chlorogenin_butyrylcholinesterase complexes, respectively.

The binding free energy was frequently calculated in the drug developing workflow using MM-PBSA (molecular mechanic Poisson-Boltzmann surface area). The free energy graph of the AChE_quercetin complex showed lower degree of fluctuation, but the other three complexes (AChE_myricetin, BuChE_alpha-amyrin, and BuChE_beta-chlorogenin) were stable and did not observe any over fluctuations. The average binding free energies of the AChE_quercetin, AChE_myricetin, BuChE_alpha-amyrin, and BuChE_beta-chlorogenin complexes were -68.798, -108.196, 79.876, and 71.315 kJ/mol, respectively (Figure 7).

### 3.6. Principal Component Analysis (PCA)

**Figure 8:**
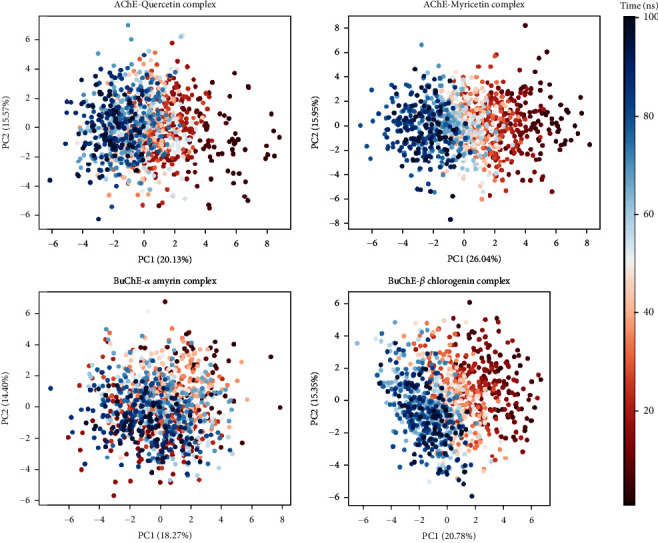
Principal component analysis (PCA). The PCA was performed to analyze the structural conformations of AChE_quercetin, AChE_myricetin, BuChE_alpha amyrin, and BuChE_beta-chlorogenin complexes using a 100 ns simulation runtime trajectory. The analysis was carried out using the scikit-learn library in Python v.3.10. The objective was to examine the variance in structural features observed during the molecular dynamic (MD) simulation, which captures the primary complex motions. In order to depict the progression of time from 1 to 100 ns, a color gradient ranging from red to white to blue was utilized for visualization purposes.

Structural data of AChE and BuChE complexes were evaluated through the principal component analysis model from MD simulation (Figure 8). For AChE_quercetin complex, a total of 35.70% of the variance was expressed wherever PC1 and PC2 expressed 20.13% and 15.57% variance, and 41.99% of the variance was exhibited by AChE_myricetin complex, wherever PC1 and PC2 expressed 26.04% and 15.95% variance, respectively. Furthermore, BuChE_alpha amyrin complex expressed a total variance of 32.67% comprising 18.27% and 14.40% of PC1 and PC2 variance, respectively. BuChE_beta-chlorogenin complex also showed a total variance of 36.13% encompassing PC1 and PC2 of 20.78% and 15.35% variance.

## 4. Discussion

Alzheimer disease (AD) is a chronic progressive neurodegenerative disease that disturbs vast area of the cerebral mantle and hippocampus leading to dementia cases [47]. On the basis of the many causal elements, several theories have been proposed to explain this complex illness like cholinergic hypothesis, AB hypothesis, tau hypothesis, and inflammation hypothesis [48, 49]. The buildup of tau protein aggregates in neurofibrillary tangles and the deposition of insoluble forms of amyloid beta (AB) in plaques in extracellular spaces and blood vessel walls are both indicators of AD [50]. The use of approved medications such as donepezil, rivastigmine, galantamine, and memantine is encouraged by medical professionals; however, these medicines only slow the spread of the illness and alleviate its symptoms, not actually cure it [48]. Computer-aided drug design can be a powerful tool for finding reliable medication candidates that can be used to fight specific AD targets.

The cholinergic hypothesis, which directly causes cognitive loss, is the most widely accepted theory for how AD develops. Furthermore, it has been discovered that the cholinesterases (ChEs), AChE, and BuChE can also contribute to the formation of amyloid protein plaques [51]. As a result, AChE inhibition and BuChE inhibition have been identified as significant targets for the efficient therapy of AD by increasing the availability of acetylcholine in brain areas and decreasing A*β* accumulation [9]. Two cholinesterases share a lot of similarities in their general structures. Each of them has a peripheral anionic site (PAS), a deep gorge, and a catalytic active site (CAS). AChE and BuChE have almost 65% homologous amino acid sequences [30].

In order to forecast the development of novel medications, a technique known as molecular docking uses a small molecule to bind to the binding site of the target receptor and demonstrate its affinity [25]. The binding affinity increases with decreasing binding energy (docking score), and vice versa. In this study, a total of 1701 ligand molecules were chosen in order to inhibit the AChE and BuChE proteins, which is responsible for the development of AD. Each of the 1701 ligands was docked against the two target receptors to evaluate their anti-AChE and anti-BuChE activity, and from this study, two best ligands from each target were selected for further analysis. The target receptor inhibition efficacy of the ligand molecules with the lowest binding energy or docking score was determined to be the best, as a lower binding energy corresponds to higher binding affinity.

This study reveals the high affinity of quercetin and myricetin towards AChE as depicted by their binding energies of -10.6 and -10.5 kcal/mol, respectively, as well as alpha-amyrin and beta-chlorogenin towards BuChE as depicted by their binding energies of -12.1 and -11.2 kcal/mol, respectively. In contrast, the donepezil revealed the affinity -7.6 kcal/mol with AChE protein and -9.8 kcal/mol with BuChE protein which indicates the lower affinity than spice plant phytochemicals.

The first compound quercetin is found to have anti-inflammatory, antiobesity [52], and cardioprotective action in cardiovascular diseases [53] and antioxidant properties [54], antiarthritic effect [55], antihypertensive effect [56], anticancer properties [57], antidiabetic effects [57], antiallergic effects [52], and so on. This phytochemical formed multiple noncovalent bonds with AChE protein and interacted with GLN71, TYR72, VAL73, ASN87, PRO88, GLY120, GLY121, TYR124, GLY126, TYR133, SER203, GLY448, TYR449, ILE451, ASP74, TRP86, SER125, GLU202, HIS447, and TYR337 amino acid residues. Myricetin contains various therapeutic potentials as anticancer [58], antioxidants and prooxidants [59], antihypertensive [60], neuroprotective [61], osteoprotective [62], antidiabetic [63], antiobesity [64], cardio-cerebrovascular protective [65], antiepileptic [66], anti-Alzheimer [66], hepatoprotective [66], gastroprotective [66], etc. activities. Myricetin interacted with GLN71, VAL73, PRO88, TYR124, SER125, GLY126, LEU130, TYR133, SER203, TYR337, TYR341, PRO446, ILE451, GLY448, TYR449, TYR72, ASP74, ASN87, GLY120, GLU202, HI+S447, GLY121, and TRP86 amino acid residues of AChE protein.

In a case, alpha-amyrin is a natural triterpenoid compound found in various plant sources. It also has several bioactive potentials like antiproliferative [67], antihyperglycemic and hypolipidemic [68], anti-inflammatory [69], analgesic [70], anticonvulsant, anxiolytic, antidepressant, gastroprotective, hepatoprotective [71], and anti-Parkinson [72] properties. Alpha-amyrin has noncovalently interacted with ASP70, GLY116, GLN119, THR120, GLU197, PRO285, SER287, ASN289, PHE329, TRP430, TYR440, GLY439, TYR332, TRP82, ALA328, and HIS438 amino acid residues of BuChE. Beta-chlorogenin interacted with ASN68, ASP70, GLY116, GLN119, THR120, GLU197, GLU276, ALA277, SER287, GLY439, ASN289, TRP82, TRP82, and HIS438 amino acid residues of BuChE protein. Beta-chlorogenin belongs to the triterpenoid group of natural products. Triterpenoids possess various bioactive properties such as anticarcinogenic [73], antidiabetic [74], anti-inflammatory [75], antimicrobial [76], antiviral [77], hepatoprotective [78], and cardioprotective [79].

When the two best ligands for each protein were compared with the positive controls, it was observed that the performances of donepezil in the docking studies were less satisfactory than quercetin and myricetin as well as alpha-amyrin and beta-chlorogenin. For this reason, it can be concluded that the best selected ligand molecules showed superior performances in the molecular docking studies. Moreover, interactions of these four ligands with the active sites of the protein residues with precise stiffness were confirmed by postmolecular dynamics, which may be the cause of the inhibition.

The selected best ligand molecules also satisfied the drug likeness criteria as predicted through the Lipinski filter and admetSAR and SwissADME analyses, which highlighted their prospective pharmacokinetic features. The drug discovery and development processes are made easier with the aid of drug likeness property estimation [80]. The topological polar surface area (TPSA) and molecular weight have an impact on how permeable a medicine is to the biological barrier. Lower permeability of the drug molecule is associated with larger molecular weight and TPSA values and vice versa. The partition coefficient of a pharmacological molecule in both the organic and aqueous phases is used to express lipophilicity (LogP). It has an impact on how quickly drug molecules are absorbed by the body, with higher LogP indicating slower absorption and vice versa. Besides, the selected ligand molecules possess the optimum level of molecular weight, number of H-bond donor, and number H-bond acceptor [81]. The two ligands for each targeted protein also followed the Lipinski five rules with minimal violation. Toxicity profiles also exhibited the absence of carcinogenicity and AMES toxicity potential, which ultimately indicates the potential of the studied compounds as being comparable to already established medicators for AD.

The PharmMapper server [82] studied the pharmacophore mapping of the best ligand compounds for each of the target protein. Three different sorts of scores are produced by PharmMapper: fit score, normalized fit score, and *z*′-score. The target proteins (receptors) with the best normalized fit scores and fit scores indicate that they should be possible binding sites for a query ligand molecule. Additionally, the fit score generates a *z*′-score, and a high *z*′-score indicates that the target is highly significant to the query compound and vice versa [83–85]. The quercetin and myricetin for AChE target protein as well as alpha-amyrin and beta-chlorogenin for BuChE protein generated the considerable fit score, normalized fit scores, and *z*′-score that should be most potential target for ligand molecules.

Quercetin, a flavonoid compound, has been studied with modifications made to different positions, such as hydroxyl groups on the B-ring and the presence of methoxy groups. These modifications have been explored for their impact on the compound's antioxidant, anti-inflammatory, and anticancer activities [86]. Specific structural features, particularly the presence of multiple hydroxyl groups on the flavonoid backbone, are associated with the ability to scavenge radicals and neutralize free radicals [87]. Changes in planar structure to induce rigidity or introduce steric hindrance can influence the compound's binding affinity and biological activity [88]. Myricetin contains several hydroxyl groups (-OH) attached to its flavonoid backbone. Modifications, such as the addition or removal of hydroxyl groups, can affect the potency and efficacy as an antioxidant [89]. Substitutions at various positions on the B-ring have been found to affect its anti-inflammatory and antiviral activities [90]. Modifications of C-ring of myricetin can also significantly impact its biological properties [91]. Glycosylation of myricetin can impact the bioavailability, stability, and cellular uptake, potentially affecting its therapeutic efficacy [92]. Conjugation and ring modification of the flavonoid rings can also influence the biological activity [93].

The presence of specific functional groups, such as hydroxyl groups or carboxyl groups, has been found to contribute to the anti-inflammatory properties of alpha-amyrin derivatives. Studies has shown the impact of functional group modifications, such as acetylation or esterification, on the anti-inflammatory, anticancer, and antidiabetic activities of alpha-amyrin [94, 95]. Additionally, specific structural features that are important for enhancing the anticancer potential of alpha-amyrin derivatives are also reported, such as modifications to the C-3 position [96]. Altering the size, configuration of sugar group can help in terms of activity and bioavailability of beta-chlorogenin [97]. Modification of aglycone core and chiral centers may provide desired pharmacological properties and features for receptor interactions [98, 99]. The length and substitution patterns of the side chains were explored to evaluate their impact on binding affinity and biological effects [100].

To support the molecular docking assessment, a molecular dynamic simulation research was conducted for the best-studied complexes, and several parameters from the simulation trajectories were evaluated to determine binding rigidity. RMSDs of all the docked complexes were under 2.5 Å. The SASA profiles for the four docked complexes did not change during the simulation trajectories for any of the four complexes, which had minimal degrees of variation. These four complexes analyze the quantitative patterns of hydrogen bonding and examine the Rg profiles that matched other simulation characteristics in many regards. The RMSF plot indicates that a little bit fluctuations of amino acid residue are present in the protein during ligand-bound state at several times. The outcomes show that the interaction of the ligand and protein reduces the distance between the two subunits (the two arms of the protein) and moves them closer to one another.

MM-PBSA analysis assesses van der Waals and electrostatic interactions, providing insights into their strength and nature, while considering solvation effects and the influence of water molecules on complex stability. By breaking down the binding free energy into these components, MM-PBSA analysis aids in identifying important interactions, evaluating the contributions of binding sites, and identifying regions suitable for ligand optimization or protein modifications [101, 102]. The negative value of acetylcholinesterase complexes suggested that the binding of the ligand to the protein is energetically favorable, and there is a likelihood of strong interactions and stability between the two molecules. The positive value of MM-PBSA value of butyrylcholinesterase complexes indicated that ligand might not form strong interactions with the protein compared to acetylcholinesterase and further MD simulation is needed.

PCA was done to examine binding cluster variance of the protein-ligand complex. Red to white to blue representing 1 ns to 100 ns simulation time were categorized into coordinates clustered in the PCA calculation by two confirmations PC1 and PC2 [103]. Both of the AChE complexes showed small variability and stable binding. AChE_quercetin complex exhibited 14.98% less variance ratio indicating its lesser conformational changes. Also, a stable minimal variance of atomic motions and Eigen scores of BuChE complexes was exhibited through the analysis. BuChE_alpha-amyrin expressed 9.58% lower variability compared to the beta-chlorogenin complex also suggesting lower changes in structural conformation [104].

**Figure 9:**
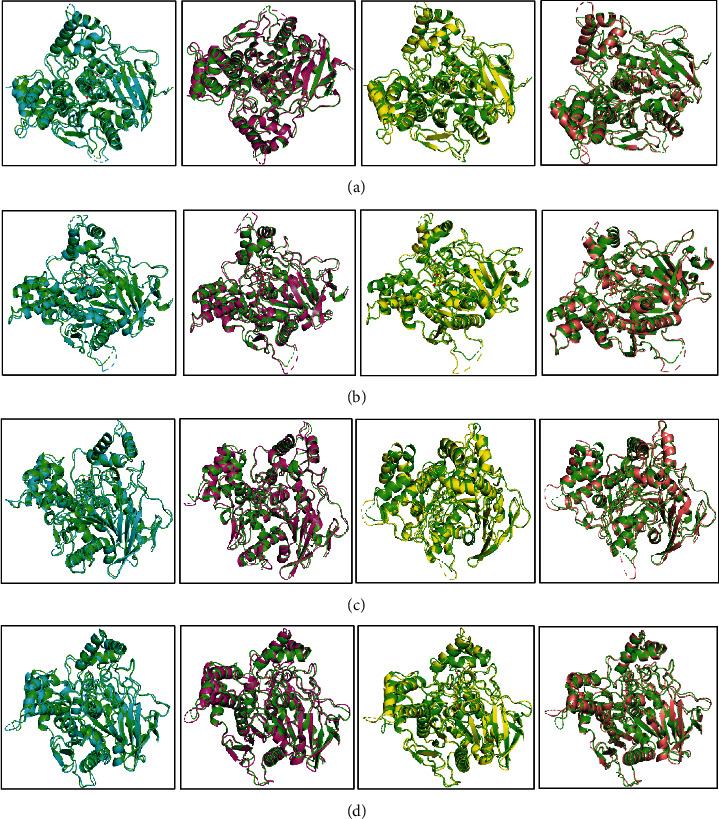
The superimposed comparison of the protein-ligand complexes after 25, 50, 75, and 100 ns simulation time: (a) acetylcholinesterase_quercetin, (b) acetylcholinesterase_myricetin, (c) butyrylcholinesterase_*α*-amyrin, and (d) butyrylcholinesterase_*β-*chlorogenin complexes. The structures obtained at 25 ns, 50 ns, 75 ns, and 100 ns are color-coded as paste, pink, yellow, and amber, respectively. This color scheme allows for an easy distinction and observation of any conformational changes or shifts in the protein-ligand complexes over the course of the simulation.

The superimposition of the structure was used to assess the binding residues and their stable interactions after taking the snapshots from 25, 50, 75, and 100 ns where the ligand molecules were found at similar binding pockets (Figure 9).

Our study demonstrated that the active compounds of ten daily used spice plants exhibit significant potential by docking with the AD target proteins AChE and BuChE. These findings point to the necessity for further in vitro and in vivo research to determine the therapeutic potential of these molecules for the secure and efficient treatment of AD.

## 5. Conclusion

In this workflow, first, we shortlisted 1701 phytochemicals from ten routinely used spices from Dr. Duke's Phytochemical and Ethnobotanical Databases. To find strong inhibitors that might be able to prevent these macromolecules' ability to catalyze reactions, these substances were tested against the AChE and BuChE proteins. Top ten lead inhibitors for each of the protein were selected according to the docking score having lower binding affinity. Through ADME/TOX analysis, two hit compounds for each protein have been evaluated biologically and pharmacologically, which supports their less harmful profile and higher drug-like qualities: quercetin and myricetin for AChE and alpha-amyrin and beta-chlorogenin for BuChE. Also, the potent docked complexes were run through molecular dynamic simulation which confirms the rigidness and stable nature of the docked complexes according to the evaluations of simulation trajectories and numerous descriptors. As this combinatorial screening was entirely reliant on computational workflows, additional in vitro experiments must be conducted to corroborate the precise targeting of these medications.

## Figures and Tables

**Table 1:** Docking results (kcal/mol)) estimated for the top two compounds and control.

Name of the targets	Name of the ligands	Binding affinity (kcal/mol)	Interacting amino acids	Bond distance in Å	Type of interaction
Acetylcholinesterase	Quercetin	-10.6	ASP74	3.73	Conventional hydrogen bond
			TRP86	6.24	Conventional hydrogen bond
				4.04	Pi-pi stacked
			SER125	3.81	Conventional hydrogen bond
			GLU202	4.21	Conventional hydrogen bond
			TYR 337	7.54	Pi-pi stacked
			HIS447	3.71	Conventional hydrogen bond
	Myricetin	-10.5	TYR72	4.18	Conventional hydrogen bond
			ASP74	3.68	Conventional hydrogen bond
			TRP86	4.02	Pi-pi stacked
			ASP87	3.24	Conventional hydrogen bond
			GLY120	4.70	Conventional hydrogen bond
			GLU202	4.66	Conventional hydrogen bond
			HIS447	4.68	Conventional hydrogen bond
	Donepezil	-7.6	LEU76	5.20	Pi-sigma
				6.19	Alkyl
			TYR124	6.74	Conventional hydrogen bond
			TRP286	5.68	Pi-pi stacked
			TYR341	4.23	Pi-sigma

Butyrylcholinesterase	Alpha-amyrin	-12.01	TYR332	4.67	Pi-sigma
			TRP82	4.49	Alkyl
				4.57	Alkyl
				4.60	Alkyl
				4.91	Pi-alkyl
				5.30	Pi-alkyl
			ALA328	7.20	Alkyl
			PHE329	5.94	Pi-alkyl
			HIS438	5.20	Pi-alkyl
				6.06	Pi-alkyl
	Beta-chlorogenin	-11.2	TRP82	4.64	Pi-alkyl
				4.84	Pi-alkyl
				4.99	Pi-sigma
				5.04	Pi-sigma
			ASP289	5.30	Conventional hydrogen bond
			HIS 438	4.73	Pi-alkyl
	Donepezil		TRP82	4.24	Pi-pi stacked
		-9.8		4.90	Pi-sigma
			TRP231	6.21	Pi-pi stacked
			LEU286	4.45	Pi-sigma

**Table 2:** Pharmacological profile of the top 10 potential candidates for AChE that were derived from SwissADME and admetSAR web server.

	Compounds	MW (g/mol)	Num. of H-bondacceptors	Num. of H-bonddonors	MlogP	Molar refractivity	TPSA (Å^2^)	AMES toxicity	Carcinogens	Violation
AChE	Fmoc-His(Trt)-Wang resin	603.71	4	1	4.64	178.48	73.22	None	None	2
	Cucurbitaxanthin A	584.87	3	2	6.15	186.28	49.69	None	None	2
	(3R,3′R,15-cis)-b,b-carotene-3,3′-diol	568.87	2	2	6.96	186.76	40.46	None	None	2
	Epsilon-carotene	536.87	0	0	8.96	184.43	0	None	None	2
	Quercetin	302.24	7	5	-0.56	78.03	131.36	None	None	0
	Quercetin-3′-glucuronide	478.36	13	8	-2.6	110.77	227.58	None	None	2
	Myricetin	318.24	8	6	-1.08	80.06	151.59	None	None	1
	Folic acid	441.4	9	6	-0.62	111.92	213.28	None	None	2
	Quercetin 3-O-glucuronide	478.36	13	8	-2.6	110.77	227.58	None	None	2
	Luteolin	286.24	6	4	-0.03	76.01	111.13	None	None	0

**Table 3:** Pharmacological profile of the top 10 potential candidates for BuChE that were derived from SwissADME and admetSAR web server.

	Compounds	MW (g/mol)	Num. of H-bondacceptors	Num. of H-bonddonors	MlogP	Molar refractivity	TPSA (Å^2^)	AMES toxicity	Carcinogens	Violation
BuChE	Proanthocyanidin A2	576.5	12	1	0.14	144.14	209.76	None	None	3
	Procyanidin B1	578.52	12	10	-0.26	146.71	220.76	None	None	3
	Alpha-amyrin	426.72	1	1	6.92	135.14	20.23	None	None	1
	Strictinin	634.45	18	11	-2.42	141.85	310.66	None	None	3
	Proanthocyanidin B2	578.52	12	10	-0.26	146.71	220.76	None	None	3
	Fmoc-His(Trt)-Wang resin	603.71	4	1	4.64	178.48	73.22	None	None	2
	Beta-chlorogenin	432.64	4	2	4.23	123.23	58.92	None	None	1
	Beta-amyrin	426.72	1	1	6.92	134.88	20.23	Yes	None	1
	Oleanolic acid	456.7	3	2	5.82	136.65	57.53	None	Yes	1
	Coriandrinonediol	458.72	3	2	5.12	136.72	57.53	None	None	1

**Table 4:** Results of the pharmacophore mapping experiment of the two best ligands for each of the protein.

Protein	Name of the compounds	Fit score	Normalized fit score	Pharmacophore features (numbers)					
				Hydrophobic center	Positively charged center	Negatively charged center	H-bond donor	H-bond acceptor	Aromatic ring
AChE	Quercetin	2.97	0.7425	2	0	0	2	0	0
	Myricetin	2.986	0.7464	2	0	0	2	0	0

BuChE	Alpha-amyrin	4	1	4	0	0	0	0	0
	Beta-chlorogenin	3	1	3	0	0	0	0	0

## Data Availability

All data are available in the manuscript.

## Additional Points

*Highlights*. (i) The distinguishable 1701 phytochemicals of routinely used ten spice plants were retrieved from Dr. Duke's Phytochemical and Ethnobotanical Databases. (ii) The molecular docking was performed against the AChE and BuChE to find a suitable drug candidate. (iii) The top ten possible drug candidates for each protein were assessed with the help of ADMET profile as well as drug likeness and were predicted two for each protein to be safe with no toxicity. (iv) The docked interactions were confirmed by the molecular dynamic simulation analysis, where rigid profiles and stable conformations of the docked complexes were observed.
